# The influence of genetic factors on the severity of anxiety and depressive symptoms and the choice of coping strategies in reproductive tract cancer―a preliminary study

**DOI:** 10.3389/fpubh.2025.1543696

**Published:** 2025-05-09

**Authors:** Anna Jurczak, Anita Chudecka-Głaz, Anna Michalczyk, Dorota Ćwiek, Joanna Owsianowska, Sylwia Wieder-Huszla

**Affiliations:** ^1^Department of Specialist Nursing, Pomeranian Medical University in Szczecin, Szczecin, Poland; ^2^Department of Gynecological Surgery and Gynecological Oncology of Adults and Adolescents, Pomeranian Medical University in Szczecin, Szczecin, Poland; ^3^Department of Psychiatry, Pomeranian Medical University in Szczecin, Szczecin, Poland; ^4^Department of Obstetrics and Pathology of Pregnancy, Pomeranian Medical University in Szczecin, Szczecin, Poland

**Keywords:** women, gynecological cancer, adaptation, depression, anxiety

## Abstract

**Objectives:**

The aim of this study was to analyze the impact of polymorphisms within the promoters of the MAO-A and the 5-HTT (SLC6A4) genes on the severity of anxiety and depressive disorder symptoms, and adaptation to the disease in patients with reproductive tract cancer.

**Methods:**

This study involved a group female patients treated at the Department of Gynecological Surgery and Gynecological Oncology of Adults and Adolescents of the Pomeranian Medical University in Szczecin. The inclusion criteria for the study were advanced ovarian cancer or endometrial cancer, as well as treatment in the form of cytoreductive therapy and chemotherapy. The following standardized research tools were used to collect empirical data: Beck Depression Inventory, State–Trait Anxiety Inventory and Mini-Mental Adjustment to Cancer.

**Results:**

The study included 139 women diagnosed with endometrial cancer (63%) or ovarian cancer (37%). Assessment of the severity of anxiety and depressive symptoms in the studied group of patients depending on genotype did not show statistically significant differences. However, among patients with genotype MAO-A 4/4, the constructive style prevailed over the destructive one, and the most frequently chosen strategy was positive redefinition. In the case of patients with the 5-HTT gene polymorphism, the most frequently chosen strategies were anxious preoccupation and positive redefinition.

**Conclusion:**

Searching for the relationship between genetic factors and the strategies adopted to cope with cancer requires intensive research. Undoubtedly, the severity of anxiety and depressive symptoms has an impact on adaptive behavior and the process of onco-logical treatment.

## 1 Introduction

Depression is a worldwide issue, and the one that is often accompanied by anxiety. The mechanism for the co-occurrence of these two has not yet been elucidated. Scientists debate whether anxiety and depression are two different conditions or different symptoms of one illness (1). Studies show that the emergence of depressive symptoms may be determined by numerous factors, among which comorbidities receive particular attention. A higher risk of depression may be directly or indirectly related to the biological, psychological and/or social effects of many diseases, including cancer (2–7). Depression and anxiety are serious medical problems also for oncology patients. An explanation for this phenomenon can be found in traditional concepts of psychopathology, which assume that symptoms of mental disorders reflect the underlying disease (8–11). Studies show that almost half of cancer survivors suffer from anxiety disorders, and approximately 15–25% of patients diagnosed with cancer have symptoms of depression (12). At the same time, depressive disorders occur in 56% of patients with anxiety, and anxiety is experienced by 47–58% of patients with a history of a depressive episode. Therefore, it is so important to simultaneously verify the presence of these two in cancer patients (8, 12). Undoubtedly, the great intensity of negative emotions promotes symptoms of anxiety and depression. Oncology patients are more likely to present the entire spectrum of emotions―mental distress, anxiety, depressive symptoms, fear, sadness, anger, aggression, a sense of guilt, and distrust. All of these are important indicators and may already appear during the diagnosis of cancer disease or later during treatment, and sometimes persist after its completion (13–16). Anxiety and depression are not destructive to all cancer patients. Some people, depending on individual psychological resources and psychosocial support, develop adaptive coping strategies for anxiety and depressive disorders (17). This is supported by the existing theories of evolutionary psychiatry, which assume that low-intensity depression and anxiety, under certain conditions, constitute an adaptive mechanism that allows a person to adopt an individual response strategy in a difficult situation (17). As confirmed by numerous studies, the risk of depression is determined by gene–environment interactions (18–25). Nevertheless, further research in this field is needed. Psychosocial factors most likely only predispose genetically susceptible individuals to depression. Among genetic factors, researchers mention the polymorphisms within the promoters of genes encoding the serotonin transporter (5-HTT) and monoamine oxidase A (MAO-A) (the enzyme catalyzing the oxidative degradation of monoamines) (26–28). A meta-analysis conducted by Sharpley’s team confirmed the interaction of the 5-HTTLPR polymorphism within the promoter region of the 5-HTT (SLC6A4) gene with stressful life events and depression (29). The aim of this study was to analyze the impact of polymorphisms within the promoters of the MAO-A and the 5-HTT (SLC6A4) genes on the severity of anxiety and depressive disorder symptoms, and adaptation to the disease in patients with reproductive tract cancer.

## 2 Methods

### 2.1 Project of the research

This survey-based study involved a group of 139 female patients treated at the Department of Gynecological Surgery and Gynecological Oncology of Adults and Adolescents of the Pomeranian Medical University in Szczecin. Informed consent was a prerequisite for participation in the study. The study was conducted in accordance with the Declaration of Helsinki, and the protocol was approved by the Bioethical Commission (Resolution No. KB-0012/81/18). The inclusion criteria for the study were advanced ovarian cancer or endometrial cancer (as these are the most common cancers of the reproductive tract in women), as well as treatment in the form of cytoreductive therapy and chemotherapy.

### 2.2 Research instruments

The study was based on a survey performed using a questionnaire technique. The following standardized research tools were used to collect empirical data:

- Beck Depression Inventory–Second Edition (BDI-II) by A. Beck, adapted by E. Łojek, J. Stańczak (30, 31),
- State–Trait Anxiety Inventory (STAI) by C. D. Spielberger, R. L. Gorsuch, R. E. Lushene, adapted by J. Strelau, M. Tysarczyk, K. Wrześniewski (32),
- Mini-Mental Adjustment to Cancer (Mini-MAC) scale by M. Watson et al., adapted by Z. Juczyński (33).

We also used an original questionnaire concerning basic sociodemographic data, i.e., age, place of residence, employment status, education, marital status and menstruation, family history of cancer, medications taken, and physical activity.

#### 2.2.1 Beck depression inventory–second edition (BDI-II)

This questionnaire is to determine depression. It consists of 21 statements that describe the most commonly observed symptoms of depression (emotional, cognitive, motivational, and physical). Items 1–14 concern the cognitive sphere (including sadness, a feeling of being worthless, pessimism, loss of pleasure, a sense of guilt, a feeling of being punished, self-criticism, suicidal thoughts, crying, loss of interest), while items 15–21 refer to the somatic realm (including loss of energy, sleep problems, appetite problems, fatigue, loss of interest in sex). Based on this scale, depression can be ruled out (0–11) or classified as mild (12–26), moderate (27–49), or severe (50–63). It is believed that in the Polish population, a score above 11 points suggests the presence of depression and is an indication for psychiatric consultation to verify the diagnosis (30, 31).

#### 2.2.2 State–trait anxiety inventory (STAI)

This questionnaire has been developed to measure anxiety. It consists of two subscales: the first of them (STAI X-1) is used to assess anxiety as a state (transient, situationally conditioned), and the second (STAI X-2) allows analysis of anxiety as a trait (understood as a relatively permanent personality trait). Each subscale consists of 20 items. The score for each part of the inventory can range from 20 to 80 points. High scores suggest high levels of anxiety. Raw scores are converted into sten scores ranging from 1 to 10, where a score of 1–4 indicates a low level of anxiety, 5–6 means a medium level of anxiety, and 7–10 reflects a high level of anxiety (32).

#### 2.2.3 Mini-mental adjustment to cancer (Mini-MAC)

The questionnaire enables the assessment of people’s adaptation to cancer and their ability to cope with the disease and its symptoms (pain, fatigue, malaise). This self-reported 29-item instrument measures the utilization of cancer-specific coping strategies, namely anxious preoccupation, fighting spirit, helplessness/hopelessness, and positive redefinition. These are grouped into two subscales: the constructive style (fighting spirit and positive redefinition), and the destructive style (anxious preoccupation and helplessness/hopelessness). Each statement is rated on a four-point scale: from 1 meaning ‘definitely does not apply to me’ to 4―‘definitely applies to me’. The score for each strategy is calculated separately and ranges from 7 to 28 points. The higher the score, the higher the utilization of a given coping strategy (33).

### 2.3 Genotyping

In accordance with the study protocol, after obtaining her consent to participate in the study each of the qualified women had venous blood collected using the Monovette closed system. DNA was isolated from the whole blood samples with the use of Invisorb Spin Blood Mini Kit (Invitec Molecular GmbH, Germany). The examined DNA regions were amplified in the PCR reaction, and then the length of the amplified fragments was analyzed using electrophoresis on 3% agarose gel with ethidium bromide staining. To analyze the 5-HTT (SLC6A4) polymorphism, the 44-bp ins-del fragment in the regulatory region of the gene was amplified with the use of forward primer 5’ GGC GTT GCC GCT CTG AAT GC 3′ and the revers primer 5′ GAG GGA CTG AGC TGG ACA ACC AC 3′. The PCR conditions were as followed: 94°C for 5 min, 30 x (94°C for 55 s, 55°C for 50 s, 72°C for 60 s), 72°C for 10 min. The VNTR polymorphism in the MAO-A promoter region was analyzed with the use of forward primer 5’ CCC AGG CTG CTC CAG AAA 3′ and the reverse primer 5′ GGA CCT GGG CAG TTG TGC 3′ and the PCR conditions: 95°C for 3 min, 34 x (94°C for 40 s, 57°C for 35 s, 72°C for 50 s), 72°C, 10 min. Four different MAO-A genotypes were identified in the study group: 3/3, 3/4, 4/4 and 4/5. However, only two women had the 4/5 genotype. Due to their small number, there was no point in conducting a separate analysis for this subgroup. Therefore, in all analyses these women were included in the group with the 4/4 genotype as a variant with a larger number of repeats compared to allele 3 carriers.

### 2.4 Statistical analysis

Statistical analysis was performed using the Statistica 13.3 software (TIBCO software inc.). According to the Shapiro–Wilk test, the distribution of most of the analyzed variables significantly deviated from the normal distribution, therefore non-parametric tests were used in all analyzes. For quantitative variables, group comparisons were done using the Mann Whitney U test, while for qualitative variables, a chi-square (χ2) test was used. The observed frequencies of particular 5-HTT and MAO-A genotypes were consistent with the Hardy–Weinberg equilibrium (*p* = 0.20 and *p* = 0.63, respectively). Statistical significance was set at *p* < 0.05, and results with *p*-values between 0.05 and 0.1 were considered as statistical trends (34).

## 3 Results

### 3.1 Characteristics of the study sample

The study included 139 women diagnosed with endometrial cancer (*n* = 87; 63%) or ovarian cancer (*n* = 52; 37%). The average age of the women was 61 ± 11 years (Me 62 years, IQR: 13, min: 34 years, max: 85 years). The table shows sociodemographic and clinical data of the study sample. The subgroups of patients selected according to genetic data did not differ significantly in terms of these factors, except for significant differences in the proportion of patients using and not using menopausal hormonal therapy (MHT) de-pending on the 5-HTT genotype, and significant differences in the proportion of patients with ovarian cancer and endometrial cancer depending on the MAO-A genotype (Table 1).

**Table 1:** Sociodemographic and clinical data of the study group divided by the 5-HTT (SLC6A4) and the MAO-A genotypes.

Parameter	Total (*n* = 139)	5-HTT				MAO-A			
		l/l (*n* = 46)	l/s (*n* = 74)	s/s (*n* = 19)	*p*	4/4 (*n* = 50)	3/4 (*n* = 69)	3/3 (*n* = 20)	*p*
Age [years] M(IQR)	62 (13)	62 (13)	61.5 (14)	64 (16)	0.64	62.5 (14)	62 (15)	62 (13)	0.57
BMI [kg/m2] M(IQR)	28 (9)	28 (6)	28 (9)	30 (13)	0.45	27 (7)	29 (8)	27 (9)	0.30
WHR M(IQR)	0.85 (0.13)	0.84 (0.11)	0.86 (0.14)	0.90 (0.15)	0.65	0.85 (0.12)	0.86 (0.13)	0.85 (0.17)	0.30
Education *n* (%)					0.50				0.58
Primary	16(12)	7(15)	8(11)	1(5)		7(14)	9(13)	0(0)	
Vocational	36(26)	12(26)	18(24)	6(32)		12(24)	19(28)	5(25)	
Secondary	60(43)	15(33)	35(47)	10(53)		21(42)	27(39)	12(60)	
Higher	27(19)	12(26)	13(18)	2(11)		10(20)	14(20)	3(15)	
Marital status *n* (%)					0.23				0.24
Single	14(10)	2(4)	11(15)	1(5)		2(4)	7(10)	5(25)	
Married	41(29)	10(22)	24(32)	7(37)		16(32)	21(30)	4(20)	
Widowed	68(49)	29(63)	30(41)	9(47)		25(50)	33(48)	10(50)	
Divorced	16(12)	5(11)	9(12)	2(11)		7(14)	8(12)	1(5)	
Place of residence *n* (%)					0.93				0.74
Village	30(22)	11(24)	16(22)	3(16)		8(16)	17(25)	5(25)	
City < 10 K	26(19)	9(20)	13(18)	4(21)		12(24)	11(16)	3(15)	
City 10–100 K	44(32)	14(30)	22(30)	8(42)		16(32)	20(29)	8(40)	
City > 100 K	39(28)	12(26)	23(31)	4(21)		14(28)	21(30)	4(20)	
Employment status *n* (%)					0.98				0.79
Employed	48(35)	14(30)	28(38)	6(32)		17(34)	23(33)	8(40)	
Unemployed	17(12)	7(15)	8(11)	2(11)		4(8)	11(16)	2(10)	
Pension	68(49)	23(50)	35(47)	10(53)		26(52)	32(46)	10(50)	
Retirement pension	6(4)	2(4)	3(4)	1(5)		3(6)	3(4)	0(0)	
Still menstruating *n* (%)	14(10)	4(9)	8(11)	2(11)	0.93	2(4)	9(13)	3(15)	0.20
Number of pregnancies *n* (%)					0.13				0.10
0	21(15)	4(9)	16(22)	1(5)		5(10)	10(14)	6(30)	
1–2	71(51)	26(57)	32(43)	13(68)		25(50)	40(58)	6(30)	
> 2	47(34)	16(35)	26(35)	5(26)		20(40)	19(28)	8(40)	
Number of births *n* (%)					0.25				0.32
0	22(16)	4(9)	16(22)	2(11)		5(10)	11(16)	6(30)	
1–2	82(59)	32(70)	38(51)	12(63)		31(62)	42(61)	9(45)	
> 2	35(25)	10(22)	20(27)	5(26)		14(28)	16(23)	5(25)	
Hormonal contraception *n* (%)					0.22				0.09
Never	119(86)	36(78)	66(89)	17(89)		47(94)	55(80)	17(85)	
Currently	0	0(0)	0(0)	0(0)		0(0)	0(0)	0(0)	
In the past	20(14)	10(22)	8(11)	2(11)		3(6)	14(20)	3(15)	
Hormone replacement therapy *n* (%)					**0.03**				0.71
Never	122(88)	35(76)	70(95)	17(89)		44(88)	60(87)	18(90)	
Currently	2(1)	2(4)	0(0)	0(0)		0	2(3)	0(0)	
In the past	15(11)	9(20)	4(5)	2(11)		6(12)	7(10)	2(10)	
Cancer type n (%)					0.83				**0.005**
Ovarian	52(37)	17(37)	29(39)	6(32)		21(42)	30(43)	1(5)	
Endometrial	87(63)	29(63)	45(61)	13(68)		29(58)	39(57)	19(95)	

Bold values means statistical significance of *p* < 0.05.

### 3.2 Correlations of the 5-HTT and the MAO-A genotypes with the severity of depressive and anxiety symptoms, and with coping strategies

The analysis did not confirm significant differences in the severity of anxiety as measured by the STAI X-1 and STAI X-2 scales depending on the 5-HTT and the MAO-A genotypes (Table 2).

**Table 2:** Associations of the 5-HTT (SLC6A4) and the MAO-A genotypes with the severity of anxiety symptoms as measured by the STAI.

STAI	5-HTT								
(N = 139)	Genotypes			Depending on genotypes					
	l/l (*n* = 46)	l/s (*n* = 74)	s/s (*n* = 19)	l/l vs. l/s + s/s		s/s vs. l/l + l/s		l/l vs. s/s	
	M (IQR)	M (IQR)	M (IQR)	Z	*p*	Z	*p*	Z	*p*
STAI X-1	7(4)	7 (4)	8(5)	0.527	0.598	0.898	0.369	−0.503	0.615
STAI X-2	5(3)	5 (4)	5(4)	1.137	0.256	0.146	0.884	0.271	0.786

	MAO-A								
	Genotypes			Depending on genotypes					
	4/4 (*n* = 50)	4/3 (*n* = 69)	3/3 (*n* = 20)	4/4 vs. 4/3 + 3/3		3/3 vs. 4/4 + 4/3		4/4 vs. 3/3	
	M (IQR)	M (IQR)	M (IQR)	Z	p	Z	p	Z	p
STAI X-1	7 (4)	7 (4)	6 (4)	−0.299	0.765	−0.288	0.773	0.066	0.948
STAI X-2	5 (3)	5 (3)	5 (2.5)	0.299	0.819	−1.497	0.135	1.289	0.197

There were no significant differences in the severity of depressive symptoms as measured by the BDI-II depending on the 5-HTT and the MAO-A genotypes (neither in the quantitative analysis―the number of points obtained, nor in the qualitative analysis―the presence of depressive symptoms vs. their absence) (Table 3).

**Table 3:** Associations of the 5-HTT (SLC6A4) and the MAO-A genotypes with the severity of depressive symptoms as measured by the BDI-II.

BDI	5-HTT								
(N = 139)	Genotypes			Depending on genotypes					
Score on the BDI	l/l (*n* = 46)	l/s (*n* = 74)	s/s (*n* = 19)	l/l vs. l/s + s/s		s/s vs. l/l + l/s		l/l vs. s/s	
	M (IQR)	M (IQR)	M (IQR)	Z	*p*	Z	*p*	Z	*p*
The presence of depressive symptoms^*^	9 (13)	6 (10)	9 (18)	0.910	0.362	1.222	0.222	−0.578	0.563
	n (%)	n (%)	n (%)	χ2	p	χ2	p	χ2	p
	18 (39)	21 (28)	8 (42)	0.869	0.351	0.677	0.411	0.050	0.956

Score on the BDI	MAO-A								
	Genotypes			Depending on genotypes					
	4/4 (*n* = 50)	4/3 (*n* = 69)	3/3 (*n* = 20)	4/4 vs. 4/3 + 3/3		3/3 vs. 4/4 + 4/3		4/4 vs. 3/3	
	M (IQR)	M (IQR)	M (IQR)	Z	*p*	Z	*p*	Z	*p*
The presence of depressive symptoms^*^	6 (9)	7 (13)	7 (10)	−0.380	0.704	0.679	0.497	0.140	0.889
	n (%)	n (%)	n (%)	χ2	p	χ2	p	χ2	p
	17 (34)	24 (35)	6 (30)	0.001	0.879	0.152	0.893	0.104	0.748

* Score on the BDI >11.

The combined analysis of both factors MAO-A and 5-HTT with the use of two-way ANOVA, with interactions did not show any significant impact of those genetic factors on depression or anxiety symptoms.

**Figure 1:**
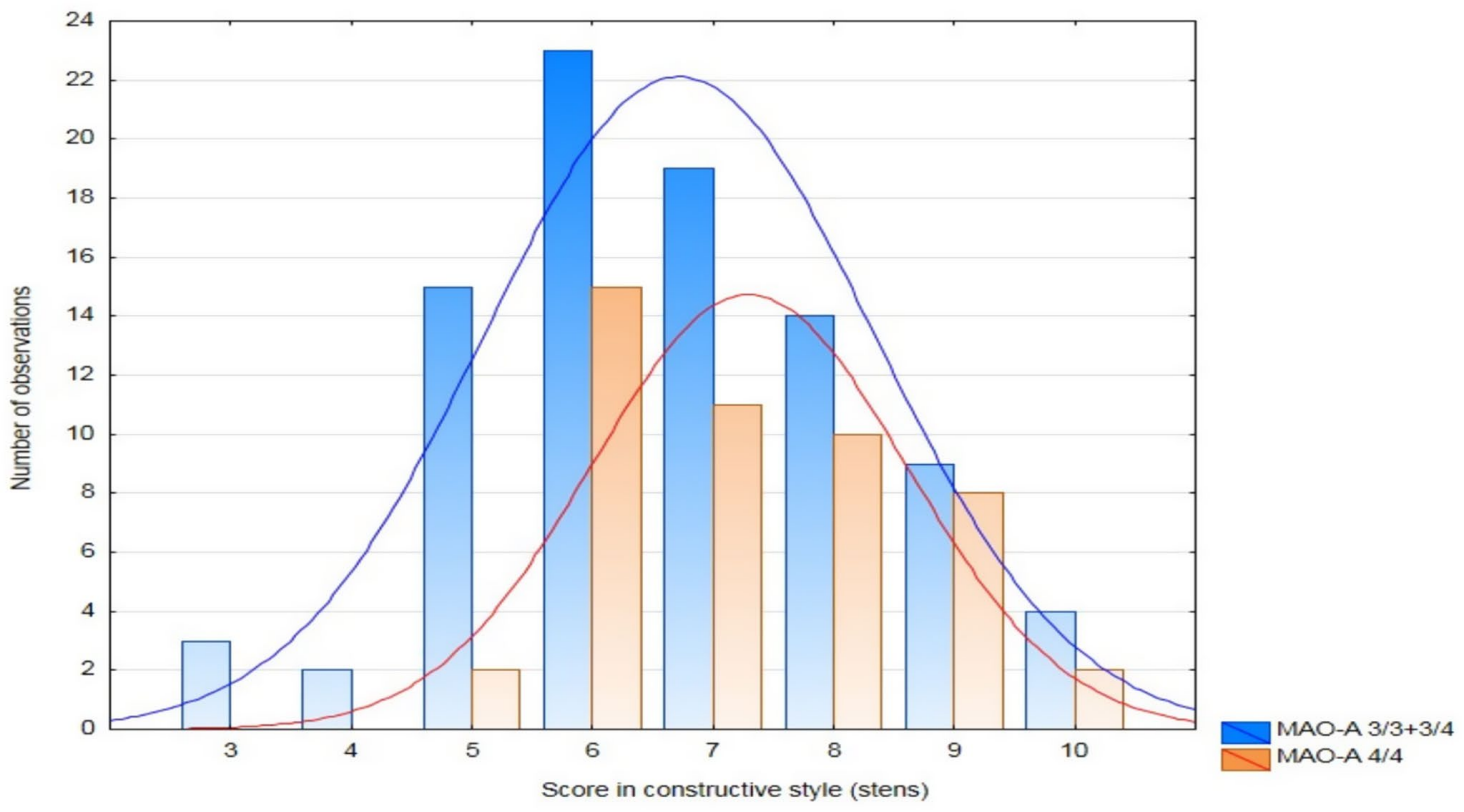
Histogram showing the distribution of the patients by sten scores for the use of the constructive style depending on the MAO-A genotype.

Patients with the MAO-A 4/4 genotype scored significantly higher for the strategy based on positive redefinition compared to the other patients (Table 4). They also obtained higher scores―both raw scores (*Z* = 2.06, *p* = 0.039) and sten scores―for the constructive style (Table 4, Figure 1). In the case of the 5-HTT gene polymorphism, patients with the s/s genotype scored higher―close to the level of statistical significance (*p* < 0.1)―for anxious preoccupation and positive redefinition than the other patients.

**Table 4:** Associations of the 5-HTT (SLC6A4) and the MAO-A genotypes with the utilization of particular coping strategies according to the Mini-MAC.

Mini-MAC	5-HTT								
(N = 139)	Genotypes			Depending on genotypes					
	l/l (*n* = 46)	l/s (*n* = 74)	s/s (*n* = 19)	l/l vs. l/s + s/s		s/s vs. l/l + l/s		l/l vs. s/s	
	M (IQR)	M (IQR)	M (IQR)	Z	*p*	Z	*p*	Z	*p*
Anxious preoccupation	18 (6)	16.5 (7)	19 (9)	1.218	0.223	1.920	**0.055**	−1.085	0.278
Fighting spirit	23 (5)	24 (6)	23 (5)	−0.250	0.803	0.065	0.948	−0.188	0.851
Helplessness/Hopelessness	11.5 (7)	13 (6)	13 (9)	−0.793	0.428	0.769	0.442	−0.869	0.385
Positive redefinition	22 (4)	21 (6)	23 (4)	0.279	0.780	1.744	**0.081**	−1.235	0.217
Constructive style (FS + PR)*	7 (2)	7 (2)	7 (2)	−0.217	0.828	0.952	0.341	−0.863	0.388
Destructive style (AP + HH)*	4 (2)	4 (3)	5 (3)	0.179	0.858	1.423	0.155	−1.034	0.301

	MAO-A								
	Genotypes			Depending on genotypes					
	4/4 (*n* = 50)	4/3 (*n* = 69)	3/3 (*n* = 20)	4/4 vs. 4/3 + 3/3		3/3 vs. 4/4 + 4/3		4/4 vs. 3/3	
	M (IQR)	M (IQR)	M (IQR)	Z	*p*	Z	*p*	Z	*p*
Anxious preoccupation	17 (8)	17 (6)	16 (9)	−0.042	0.967	0.195	0.845	−0.169	0.866
Fighting spirit	25 (6)	23 (5)	24 (6.5)	1.289	0.198	−0.371	0.711	0.772	0.440
Helplessness/ Hopelessness	13 (6)	13 (6)	11 (8)	0.713	0.476	−0.247	0.805	0.496	0.620
Positive redefinition	23 (4)	22 (4)	21.5 (6)	2.265	**0.024**	0.036	0.971	0.719	0.472
Constructive style (FS + PR)*	7 (2)	6 (2)	7 (3)	2.073	**0.038**	0.058	0.954	0.671	0.502
Destructive style (AP + HH)*	4.5 (3)	4 (3)	4 (3)	0.256	0.798	0.088	0.930	0.013	0.989

* Sten scores for particular strategies (33).

Bold values means statistical significance of *p* < 0.05.

The higher sten scores obtained by patients with the MAO-A 4/4 genotype for the constructive style are shown in Figure 1.

**Figure 2:**
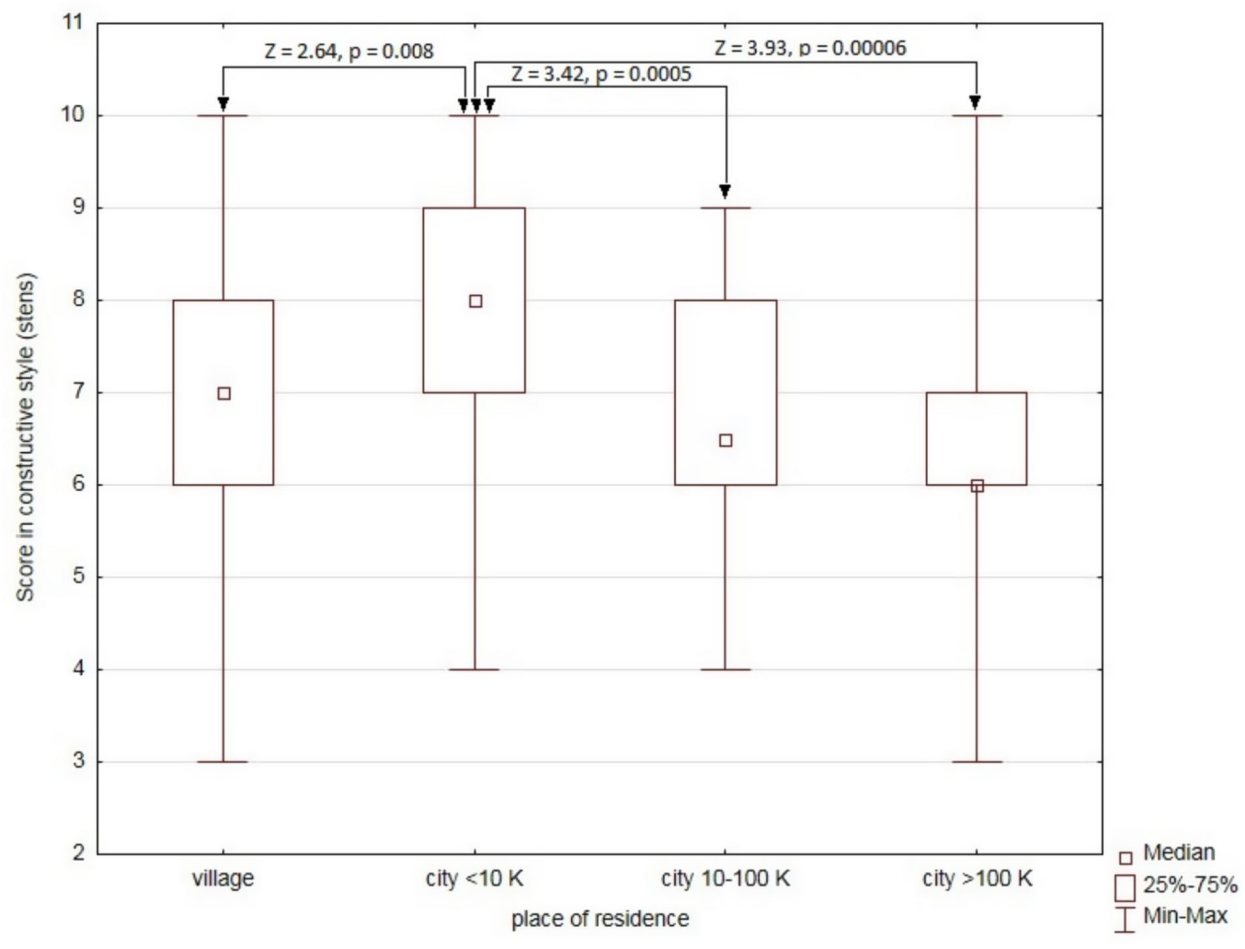
Comparison of sten scores for the use of the constructive style depending on the place of residence.

Analysis of sociodemographic and clinical factors potentially affecting the use of the constructive style (sten scores) revealed only one significant relationship―with place of residence (H = 18.17, *p* = 0.0004). Patients living in cities with up to 10,000 inhabitants were characterized by higher sten values for the constructive style than the other groups (Figure 2).

A two-way ANOVA was performed to analyze the effect of the MAO-A 4/4 genotype and place of residence on the score for the use of the constructive style. The analysis did not reveal a statistically significant influence of the interaction between the MAO-A 4/4 genotype and place of residence (F (3) = 0.48, *p* = 0.70). However, a simple analysis of main effects showed that both the MAO-A 4/4 genotype and place of residence had a statistically significant independent effect on the score for the constructive style (F (1) = 4.13, *p* = 0.044 and F (3) = 12.51, *p* = 0.0006, respectively).

## 4 Discussion

Depression and anxiety are common comorbidities in cancer patients. Undoubtedly, they exacerbate emotional stress, which reduces the quality of such patients’ lives. Therefore, emotional distress is considered an important indicator in the treatment of cancer (35). Negative emotions are experienced by patients already at the time of cancer diagnosis as well as during treatment. It has been observed that the intensity of negative emotions is associated with the frequency of experiencing symptoms of anxiety and depression (13–16). The severity of these symptoms, on the other hand, may depend on many factors, including age, sex, education, type of oncological disease, its stage, time of diagnosis, treatment methods (radiotherapy or chemotherapy), and prognosis (36). Other factors that determine the experience of anxiety and depression in this group of patients are emotional and social support, coping strategies, and the availability of therapy and information about the disease (37). In the long run, the coexistence of depressive and anxiety disorders affects the results of the therapy, and causes that disease symptoms are felt more strongly (38). It has been indicated for many years that psychosocial factors play a key role in the etiopathogenesis of depression. According to Landowski, the emergence of depressive symptoms is a result of stress and the interaction of environmental and genetic factors (39). Thus, combination of psychosocial and genetic risk factors predispose to depression. Interactions between 5-HTTLPR and stressful events experienced both in the past and recently are an important factor in depressive disorders (40, 41). The importance of the interaction between genetic and environmental factors that increase susceptibility to the disease has been confirmed by Caspi et al. (26) and Kendler et al. (42). According to these researchers, increased sensitivity to stress may be associated with the 5-HTTLPR polymorphism (26, 42). The occurrence of depressive symptoms has also been found to be influenced by the MAO-A polymorphism and by environmental factors. According to Cicchetti et al. (27) the severity of depression in the group of traumatized patients correlated with low MAO-A activity, while those with high MAO-A activity were characterized by better-developed stress coping mechanisms and less severe depressive symptoms (27).

The results of numerous studies show that individual response to stress is determined by genetic susceptibility. In the case of the 5-HTTLPR gene, significantly more severe depressive symptoms were observed in people with the short ‘s’ allele (SS or SL genotypes) than in those with the long ‘l’ allele (LL genotype) (26, 43, 44). Stein et al. (45) studied the link between the presence of the 5-HTTLPR polymorphism, depressive symptoms as measured by the BDI, and the subjects’ personality. The results did not reveal a statistically significant relationship between these variables, but suggested that the 5-HTTLPR polymorphism had an impact on the subjects’ psychological resilience (as it is directly related to the risk of anxiety and mood disorders)―people with the ‘s’ allele of this gene had higher levels of anxiety (45). In our study, an attempt to assess the level of anxiety and depressive symptoms depending on the genotype did not confirm statistically significant differences. Perhaps this was due to the fact that our study sample was too small to detect weak relationships or that different parameters were analyzed. Undoubtedly, expanding the sample size by including additional genotypes and parameters would increase the importance of the research.

Undoubtedly, coping strategies have a significant impact on the treatment process in cancer (46, 47). A large proportion of cancer patients at various stages of disease progression are diagnosed with depressive disorders, which hinder psychological adjustment to cancer (48, 49). The patient’s adoption of the constructive or destructive coping strategies may affect both their quality of life and the distant effects of the treatment. While the attitudes of fighting spirit and denial of the disease may contribute to higher survival rates, the attitudes of stoic acceptance or helplessness/hopelessness may impede the fight against the disease and disrupt defense mechanisms (47, 50, 51). Schillani’s team studied the correlation between the 5-HTTLPR polymorphism and psychological adjustment to breast cancer. The results indicated that in patients with early breast cancer, the strategies of helplessness/hopelessness and anxious preoccupation significantly correlated with depression, while avoidance correlated with anxiety. Patients with advanced cancer showed similar correlation results, and a negative correlation of depression with fighting spirit and avoidance. A significant correlation was found between helplessness/hopelessness and depression in early-stage breast cancer patients with the long L/L allele. Thus, the 5-HTTLPR polymorphism determines coping strategies and the occurrence of depression, which may have implications for further treatment (52). In our study, correlation analysis between the utilization of individual stress coping strategies and the 5-HTTLPR polymorphism showed no significant differences, except for higher values (close to statistical significance) for the strategies of anxious preoccupation and positive redefinition in patients with the s/s genotype. Patients with the MAO-A 4/4 genotype scored considerably higher on the positive redefinition strategy compared to other patients, and scored higher for the constructive style. These results indicate a potential link between genetic factors and strategies, but require confirmation on a larger group of patients.

The next stage of our research was an attempt to assess the influence of sociodemographic and medical variables on the choice of a specific strategy of adaptation to the disease. The analysis revealed only one significant association, namely the influence of place of residence on the choice of the constructive style by the respondents. It was observed that both the presence of the MAO-A 4/4 genotype and place of residence had a statistically significant independent impact on the score for the use of the constructive style. This style consists of the strategies of fighting spirit and positive redefinition, which determine treating the disease as a challenge, and thus taking action to combat it (53). An attempt to evaluate the influence of selected sociodemographic and medical variables on the degree of adaptation to the disease in a group of women treated for gynecological cancer was made by Kupcewicz’s team. The results showed that the women tended to adopt constructive ways of coping with cancer, and the age of the subjects was a determining factor (54). The literature lacks analyses of the relationship between coping strategies and place of residence. This topic seems interesting, but requires further investigation.

Oncological disease has a number of consequences, both in terms of physical and emotional functioning. Among them, the most common conditions are anxiety and depression, which are most often destructive. Therefore, it is worth taking steps to prevent and effectively treat these conditions in cancer patients.

The premise in our research was the long-term follow-up of patients with endometrial cancer and ovarian cancer. However, during the collection of the material, we encountered some limitations due to the SARS-CoV2 pandemic and subsequent sanitary-epidemiological restrictions, which reduced the size of the study sample and thus the statistical power of the results obtained. Therefore, we see the need for further research in this area with longitudinal studies conducted on a larger sample with additional genotypes and assessing the interaction between genes and other determinants of anxiety and depression.

## 5 Conclusion

To sum up, although no association was found between the 5-HTT and the MAO-A genotypes and the severity of anxiety and depressive symptoms in oncology patients, our findings showed that the dominant style among patients with the MAO-A 4/4 genotype was the constructive style, and the dominant strategy was positive redefinition.

## Data Availability

The original contributions presented in the study are included in the article further inquiries can be directed to the corresponding author.
